# Identification, Characterization and Function of Orphan Genes Among the Current Cucurbitaceae Genomes

**DOI:** 10.3389/fpls.2022.872137

**Published:** 2022-05-04

**Authors:** Dongna Ma, Zhengfeng Lai, Qiansu Ding, Kun Zhang, Kaizhen Chang, Shuhao Li, Zhizhu Zhao, Fenglin Zhong

**Affiliations:** ^1^College of Horticulture, Fujian Agriculture and Forestry University, Fujian, China; ^2^College of the Environment and Ecology, Xiamen University, Fujian, China; ^3^Subtropical Agricultural Research Institute, Fujian Academy of Agriculture Sciences, Fujian, China

**Keywords:** Cucurbitaceae, orphan genes, transcriptome, male sterility, environmental adaptation

## Abstract

Orphan genes (OGs) that are missing identifiable homologs in other lineages may potentially make contributions to a variety of biological functions. The Cucurbitaceae family consists of a wide range of fruit crops of worldwide or local economic significance. To date, very few functional mechanisms of OGs in Cucurbitaceae are known. In this study, we systematically identified the OGs of eight Cucurbitaceae species using a comparative genomics approach. The content of OGs varied widely among the eight Cucurbitaceae species, ranging from 1.63% in chayote to 16.55% in wax gourd. Genetic structure analysis showed that OGs have significantly shorter protein lengths and fewer exons in Cucurbitaceae. The subcellular localizations of OGs were basically the same, with only subtle differences. Except for aggregation in some chromosomal regions, the distribution density of OGs was higher near the telomeres and relatively evenly distributed on the chromosomes. Gene expression analysis revealed that OGs had less abundantly and highly tissue-specific expression. Interestingly, the largest proportion of these OGs was significantly more tissue-specific expressed in the flower than in other tissues, and more detectable expression was found in the male flower. Functional prediction of OGs showed that (1) 18 OGs associated with male sterility in watermelon; (2) 182 OGs associated with flower development in cucumber; (3) 51 OGs associated with environmental adaptation in watermelon; (4) 520 OGs may help with the large fruit size in wax gourd. Our results provide the molecular basis and research direction for some important mechanisms in Cucurbitaceae species and domesticated crops.

## Introduction

Genetic variation is the basis for the genetic diversity of living organisms. Genetic diversity causes the gene content of genomes to vary in different lineages (Long et al., 2003). The study of lineage-specific genes has generated great interest because these genes are particularly important in driving organisms to complete life processes such as species differentiation and adaptation to new environments (Cui et al., 2015). A special kind of lineage-specific gene is orphan genes (OGs), which represent a set of genes that are unique to a species and have no recognizable homologs to other species but encode proteins (Fischer and Eisenberg, 1999). The development of large-scale sequencing technologies has made OGs research a hotspot in comparative genomics and the analysis of large numbers of genomes has suggested that OGs are widely present in all areas of life, such as microorganisms (Yin and Fischer, 2006, 2008), plants (Campbell et al., 2007; Yang et al., 2009), primates (Lindskog et al., 2014) and insects (Sun et al., 2015).

Orphan genes are an enigmatic part of the genome that do not have any obvious “ancestor”, but play essential roles in the generation of novel functions and even phenotypic changes (Kaessmann, 2010). Studies on the *Tribolium castaneum* have revealed that embryo development was closely associated with two OGs, *Tc-flipflop1* and *Tc-flipflop2*. When these two genes were knocked down, it resulted in larval malformation (Thümecke et al., 2017). One *Arabidopsis thaliana* OG (Qua-Quine Starch, *QQS*) can affect the protein composition by influencing the process of carbon and nitrogen segregation between proteins and carbohydrates (Li et al., 2015). A secreted protein encoded by an OG in the Hydra promoted the growth of tentacles (Khalturin et al., 2008). In addition, OGs are given new biological functions that allow species to adapt to the lineage-specific environment (Tautz and Domazet-Lošo, 2011). For example, OG (*flightin*) in *Drosophila* enhanced the flight power of both wings and improves survival adaptability (Domazet-Loso and Tautz, 2003). A wheat OG (*TaFROG*) enhanced its resistance to *Fusarium* head blight (Perochon et al., 2015). Most of the 1,926 OGs identified in the rice genome were expressed more readily than other non-orphan genes (NOGs) when subjected to external environmental stresses (Guo et al., 2007), a phenomenon that also occurred in *Arabidopsis thaliana* when subjected to abiotic stresses such as oxidation or osmosis (Luhua et al., 2008, 2013; Knowles and McLysaght, 2009).

Studies found that the OGs are more inclined to be expressed in the male reproductive system. For example, twenty-seven human OGs of *de novo* origin were studied and the results showed that they are expressed mainly in the testes (Wu et al., 2011). In wheat, the *Ms2* gene encoded an orphan protein that causes male-sterility as well as male sterility in *Hordeum vulgare* and *Brachypodium Beauv* (Ni et al., 2017). These studies show the importance of the OGs for improving male reproductive fitness. In summary, OGs have a wide range of functionalities and they can participate in various regulatory pathways or metabolic pathways affecting all parts of the living organism.

The Cucurbitaceae family is the second-largest vegetable family and has among the most genetically diverse groups of plants (Schaefer and Renner, 2011). Members of this family are widespread in the tropics, and many of them are now grown as food crops around the world (Hunsakunachai et al., 2019), such as cucumber (*Cucumis sativus*), melon (*Cucumis melo*), watermelon (*Citrullus lanatus*), bottle gourd (*Lagenaria siceraria*), wax gourd (*Benincasa hispida*), pumpkin (*Cucurbita moschata*), chayote (*Sechium edule*), and snake gourd (*Trichosanthes anguina*). Despite being monophyletic, these species show intriguing phenotypic variation in fruit characters. In the last decade, the reference genomes of these Cucurbitaceae species have been deciphered due to the rapid advances of sequencing technologies and bioinformatics algorithms (Garcia-Mas et al., 2012; Sun et al., 2017; Guo et al., 2019; Li Q. et al., 2019; Ma L. et al., 2020). It became possible to detect OGs in the Cucurbitaceae genome using comparative genomics. Based on this, we identified OGs in eight Cucurbitaceae species, analyzed and compared their origin mechanisms, structural features, subcellular localization, and chromosomal distribution. Using abundant and reliable RNA-seq data, we also profiled the expression patterns of these identified OGs in different tissues and under different abiotic stresses. Finally, we constructed a weighted gene co-expression network analysis (WGCNA) and Fuzzy c-means clustering analysis to predict the potential functions of OGs. Overall, these results not only provide a valuable resource for studying the evolution and species of Cucurbitaceae, but it also provides essential molecular information for genetic studies and the improvement of Cucurbitaceae.

## Materials and Methods

### Data Sources

In this study, the eight Cucurbitaceae plant species, watermelon, bottle gourd, chayote, cucumber, melon, pumpkin, snake gourd, wax gourd, were used to identify OGs, respectively. The Cucurbitaceae genomes and annotation information were downloaded from the CuGenDB database^1^. The assembled unique transcripts (PUT) from plant mRNA sequences were downloaded from PlantGDB^2^, other 125 plant genome predicted proteins were downloaded from Phytozome^3^. UniProtKB was downloaded from Uniprot^4^ and NR database were downloaded from NCBI^5^, respectively.

To predict the potential functions of OGs, we downloaded published RNA-seq data to obtain the gene expression levels. These data included different tissues or different abiotic stresses of melon, cucumber and watermelon. We downloaded the transcriptome data of watermelon from NCBI^6^ under project number accession PRJNA454040 (leaf imposed to drought stress), PRJNA770012 (root imposed to osmotic stress) and PRJNA422970 (leaf and root imposed to different levels of nitrogen). The other RNA-seq resources of Cucurbitaceae plant species included melon (PRJNA383830: root, leaf, male flower, female flower, and fruit) and cucumber (PRJNA80169: root, stem, leaf, male flower, and female flower; PRJNA307098: Flower opening process, including green bud, green-yellow bud, yellow bud, and flowering).

### Identification of Orphan Genes

**FIGURE 1:**
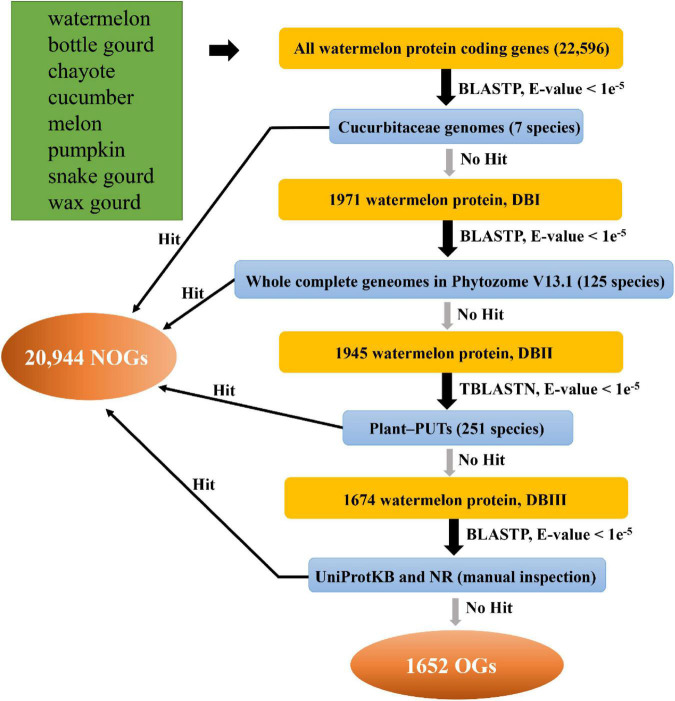
Procedures for identifying orphan genes in eight Cucurbitaceae species.

We used the comparative genomics to detect OGs in the eight species from Cucurbitaceae. The identification pipeline is shown in Figure 1. For example, we first performed a BLASTP on the watermelon protein sequences against the proteome data of the other seven collected Cucurbitaceae plants. It was discarded if the watermelon protein sequence has a significant BLASTP hit in other species with an E-value < 1e^–5^. We then performed a homolog search against Plant-PUTs database, other published plant genome sequences, UniProtKB database and NR database with an E-value < 1e^–5^, respectively. Ultimately, the genes that match to neither other databases are the OGs in watermelon (Zhang et al., 2007; Lin et al., 2010), and genes with at least one homolog are NOGs. The other seven Cucurbitaceae species were performed utilizing the same identification process.

### Genic Characterization

To analyze the characteristics of OGs in Cucurbitaceae, we downloaded the whole genome information of eight Cucurbitaceae species (see text footnote 1). Then in-house python scripts were used to calculate protein length, GC content, exon length, and the number of exons per gene^7^. The isoelectric point of the proteins was calculated using the DAMBE7 software (Xia, 2018). We used the Wilcox rank sum test to estimate significant differences between OGs and NOGs in different groups. BUSCA (Bologna Unified Subcellular Component Annotator) was then used to predict the subcellular localizations of OGs with eukarya plants mode (Savojardo et al., 2018). The chromosomal localization information of OGs was extracted from the annotation file, and then mapping was performed with Mapgene2chrom^8^.

### Origin of the Orphan Genes

The study of how genes arise and the differentiation process is essential to explain the generation and evolution of new phenotypes and finally the inheritance of biodiversity (Long et al., 2003). According to previous studies, there are four main mechanisms that account for how OGs emerged, including gene duplication, gene overlap, transposable element (TE) exaptation and *de novo* origin (Wu et al., 2011; Wissler et al., 2013). Among the four mechanisms, gene duplication is thought to be the predominant mechanism of origin (Zhang, 2003). We used DupGen_finder.pl to identify OGs originating from gene duplication, which can identify five different duplication modes, including tandem duplication, whole genome duplication (WGD), dispersed duplication, transposon duplication, and proximal duplication (Qiao et al., 2019). First, we aligned the protein sequences within a genome by BLASTP with an *E*-value < 1e^–8^. Next, DupGen_finder.pl was used to determine the model of gene duplication based on the detected homologous gene pairs. Synonymous sites (*Ks*) were computed using the Nei–Gojobori approach implemented in the python script synonymous_calc.py^9^. Finally, the universal mutation rate of 6.5 × 10^–9^ was used to assess the time of gene duplication of OGs (Gaut et al., 1996). To identity overlapping gene models, we used OGs against CDS sequences of other Cucurbitaceae species to screen for homologous sequences covering at least 50% of the length of gene. To identify the OGs overlapped with TEs, we first used the software RepeatMasker to identify TEs in eight Cucurbitaceae species, composition of a Cucurbitaceae TEs dataset (Mendeley Data)^10^ and then the CDS sequences of the Cucurbitaceae OGs were used as queries to do BLASTN searches against the Cucurbitaceae TE sequences dataset with an *E*-value < 1e^–5^. For *de novo* originated genes, orphan proteins sequences of the remaining were then searched with TBLASTN against the genomes of seven other Cucurbitaceae to identify orthologous non-coding sequences. The orthologous non-coding sequence was defined according to three indicators: (1) at least 60% sequence identity and covering at least 80% of the orthologous regions of target gene could be aligned; (2) the alignment significance *E*-value < 1e^–6^; (3) the protein lengths of other Cucurbitaceae species that have premature translational termination should be shorter than 50% of the length of the candidate orphan proteins (Wu et al., 2011; Sun et al., 2015; Zhang et al., 2019).

### Gene Expression Analysis

To further explore the role of OGs in Cucurbitaceae, we calculated the expression levels of genes in different tissues and identified differentially expressed genes (DEGs) in different treatments on the basis of the collected transcriptome data. We filtered RNA-seq data using Trimmomatic. RSEM was used to compute FPKM (fragments per killobase of exon per million fragments mapped) values. DEGs analysis was carried out with the DESeq2 R package (Love et al., 2014). The significant differences in gene expression were determined using a false discovery rate (FDR) set at < 0.05 and | log_2_FC| > 1 as cutoffs. Genes with FPKM value > 0.02 were assumed to have been expressed (Ma S. et al., 2020). Besides, PaGeFinder software with specificity measure (SPM) was used to identify the genes specifically expressed in a certain tissue (Pan et al., 2012), and it was determined as a specific gene in this tissue once the SPM value was ≥ 0.9.

### Weighted Gene Co-expression Network Analysis and Function Annotation

The co-expression network construction was completed using the WGCNA software package of R software. First, the data was filtered to construct the expression matrix and the samples were clustered for analysis. The soft threshold β function in WGCNA was used to calculate the coefficient of similarity among all genes to create the adjacency matrix and construct the systematic clustering tree. The minimum number of genes per module was set to 30, and the initial co-expression module was identified using cutreeDynamic. Subsequently, the eigenvector value ME (Module eigengene) of each module is calculated using the moduleEigengene function. The correlation coefficient between module ME and sample features is quantified using the cor function and a heat map is drawn. Higher correlation coefficients indicate a higher correlation between the genes in the module and the sample features, and accordingly, higher relative gene expressions. Modules with high correlation coefficients with sample characteristics are selected as the tissue-specific module. The module membership (MM) and gene significance (GS) of ME in each tissue-specific module were calculated. If MM > 0.95 and GS > 0.85, the gene was decided as the central gene of the module. In addition, we also used Fuzzy c-means clustering to analyze the transcriptome time series data (Parker and Hall, 2014). KEGG pathway enrichment analysis based on the KEGG biology pathway database and mapping was performed on OmicShare^11^, which is an online platform.

## Results and Discussion

### Identification of Orphan Genes Among Different Cucurbitaceae Species

Based on previous studies, we designed a comprehensive, systematic computational pipeline to identify OGs in the Cucurbitaceae genomes (Figure 1; Doerks et al., 2002; Tay et al., 2009; Yang et al., 2013; Xu et al., 2015). For instance, in the watermelon, there were 22,596 annotated protein-coding genes, which were used for BLASTP with the Cucurbitaceae genome. The 1971 genes (DatabaseI, DBI) were kept for follow-up analysis. The retained genes were then against with 125 plant genomes, and 1,945 genes had no match (DBII). In the next comparison of these genes with 251 PlantGDB-assembled Unique Transcripts (PUTs) sequences, 1674 genes were found to be non-homologous (DBIII). A final step to remove further the impact of false positives on the analysis was to analyze the remaining genes in comparison to the UniProtKB and NR databases, resulting in 1,652 genes being left. The final leftover 1,652 genes were termed as OGs in the watermelon genome (Supplementary Table 1). Using the same pipeline, we identified OGs for each of the other seven Cucurbitaceae species’ genomes (Supplementary Table 1). OGs contents greatly varied among the eight Cucurbitaceae species, ranging from 1.63% in chayote and 16.55% in wax gourd (Supplementary Table 2). The proportions of OGs in the different species varied greatly, typically in the range of 5-15%. Previous studies of sweet orange (Xu et al., 2015), rice (Guo et al., 2007), wheat (Ma S. et al., 2020), and *Populus trichocarpa* (Guo, 2013) reported 3.54, 3.23, 1.4, and 14.32% orphans, respectively. In two close relative species, they also showed great differences, the proportion of OGs in *Arabidopsis thaliana* is 5.3%, while it is 12.3% in *Arabidopsis lyrate* (Guo, 2013). Part of this variation is due to the different evolutionary distance that exists between each focal species and its closest sequenced relatives (Wissler et al., 2013). The more genomes of reference species are decoded, the more annotation messages are available, and the accuracy of prediction may become higher. Another part variation may be due to real differences in evolutionary pressures (Arendsee et al., 2014).

### Distinctive Gene Structure of Orphan Genes

**FIGURE 2:**
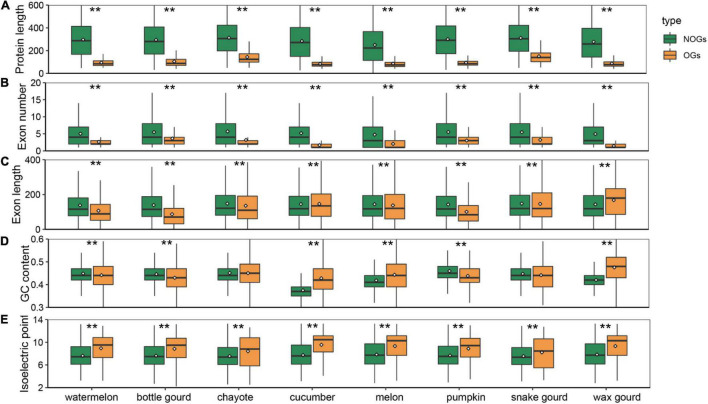
Box plot comparison of protein length **(A)**, exon number **(B)**, exon length **(C)**, GC content **(D)**, and isoelectric point **(E)** of orphan genes (OGs) and non-orphan genes (NOGs) for eight Cucurbitaceae species. Statistical analysis was performed using the Wilcox rank sum test. Statistical significance: ** *P*-value < 0.001.

Among all species, OGs have a shorter origination time, and whether they have distinctive features relative to NOGs is an intriguing question. To determine the differences that exist in Cucurbitaceae species, we performed an analysis and compared the gene structure of OGs and NOGs. Our results showed that OGs exhibit significantly shorter protein lengths in all eight species of Cucurbitaceae (Figure 2A). The average protein length distribution of the OGs and NOGs were 48-155, 348-436 amino acids, ranging from 2.78 times in snake gourd 7.25 times in melon (Supplementary Table 3, Wilcox rank sum test, *P*-value < 0.001). This is a finding that is consistent with the sequence characteristics that have been reported in primates (Toll-Riera et al., 2009), zebrafish (Yang et al., 2013), sweet orange (Xu et al., 2015), and *Caenorhabditis elegans* (Zhang et al., 2019). In-depth analysis of the structural components of the genes showed that the shorter protein length was mainly attributed to the fewer number of exons (Figure 2B and Supplementary Table 3, Wilcox rank sum test, *P*-value < 0.001). In the zebrafish, about 28% of the OGs have only one exon, compared to the NOGs with only one exon is 6% (Yang et al., 2013). About 36.87% of the genes in the maize were intronless genes, of these, about 16.67% were OGs (Yan et al., 2014). In primate species, OGs also contained fewer exons compared to NOGs (Toll-Riera et al., 2008). This suggests the prevalence of these two characteristics for OGs in all eukaryotes. We also analyzed the exon lengths of OGs and found it was significantly longer in snake gourd, while it was significantly shorter in other Cucurbitaceae species (Figure 2C and Supplementary Table 3, Wilcox rank sum test, *P*-value < 0.001). In sweet orange, the exon length of OGs was shorter than that of NOGs, but in wheat *Caenorhabditis elegans*, the exon lengths played an insignificant role (Xu et al., 2015; Li G. et al., 2019; Ma S. et al., 2020). The reason for this is the evolutionary process of short new genes to long old genes involving mainly the recruitment of alternative exons rather than the expansion of individual exon lengths (Neme and Tautz, 2013). The GC content of some species increases progressively across the phylostrata (Donoghue et al., 2011; Wissler et al., 2013), and the youngest gene in *Arabidopsis thaliana* also had a sharply separated from non-genetic ORFs (43% median GC content for OGs and 32% median GC content for NOGs) (Lin et al., 2010). However, this characteristic is not universal. In *Strobilanthes cusia*, there was no difference in GC content (Hu et al., 2021). In *Aegiceras corniculatum* and wheat, OGs were significantly less than that of NOGs (Ma et al., 2021). Among Cucurbitaceae species, they also exhibited heterogeneously. In chayote and snake gourd, the GC content of OGs was not different compared to NOGs, and in cucumber, melon and wax gourd, OGs were significantly higher, while in watermelon, bottle gourd and pumpkin, significantly lower (Figure 2D and Supplementary Table 3, Wilcox rank sum test, *P*-value < 0.001). Changes in the isoelectric point are important indicators of altered protein function (Khaldi and Shields, 2011). We found that the isoelectric points of OGs were significantly higher than NOGs in all species of Cucurbitaceae (Figure 2E and Supplementary Table 3, Wilcox rank sum test, *P*-value < 0.001), which may be associated with the fact that species have to adapt to variable environments (Andrade et al., 1998; Kiraga et al., 2007).

### Subcellular Localization and Chromosome Distribution

**FIGURE 3:**
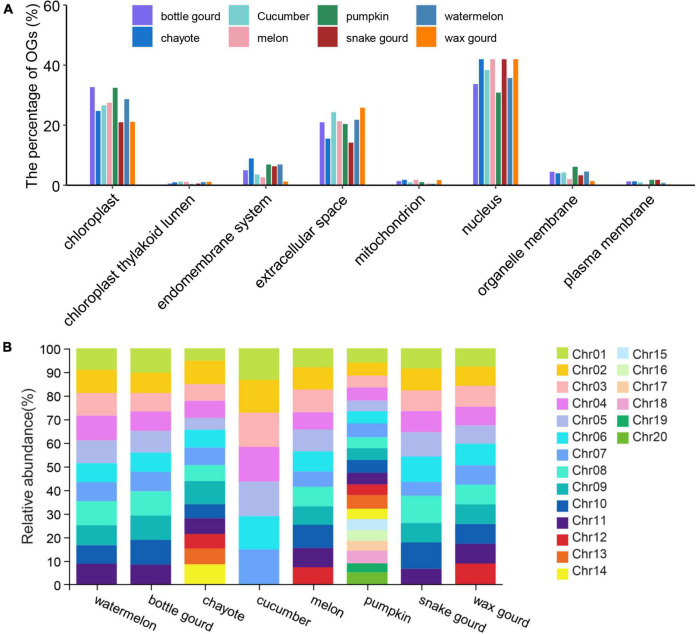
Subcellular localization **(A)** and chromosome distribution **(B)** of eight Cucurbitaceae species.

Predicting the subcellular localization is important for understanding the nature and function of proteins in cells and exploring the interactions between proteins (Emanuelsson et al., 2007; Chou and Shen, 2008). We performed subcellular localization analysis on eight Cucurbitaceae species, and the results showed that the localization was mainly on the nucleus (pumpkin: 30.86% ∼ snake gourd: 52.4%) and chloroplast (snake gourd: 20.96% ∼ bottle gourd: 32.64%) (Figure 3A and Supplementary Table 1). Except for pumpkin, which was mostly localized on chloroplast, the other seven species were mostly localized on the nucleus. In general, the subcellular localization of Cucurbitaceae species was essentially the same, with only subtle differences.

The distribution properties of OGs on chromosomes differ between species. In zebrafish, the distribution of OGs on chromosomes was heterogeneous, with a high proportion of OGs on some chromosomes and none on others (Yang et al., 2013). The OGs in the wheat genome were evenly distributed on the 21 chromosomes, with a higher density in the regions near the telomeres (Ma S. et al., 2020). OGs in the *Arabidopsis thaliana* genome were not grouped but uniformly spread across the genome among NOGs (Donoghue et al., 2011). For the analysis of OGs’ genomic distribution, we plotted the OGs over the chromosomes of Cucurbitaceae based on the available information from genome annotation (Supplementary Table 1). In watermelon, the proportion of OGs distributed on 11 chromosomes ranged from 6.33% to 8.30%, bottle gourd (3.35% ∼ 4.42%), chayote (1.65% ∼3.23%), cucumber (8.85% ∼9.85%), melon (5.59% ∼8.5%), pumpkin (5.79% ∼9.22%), snake gourd (1.30% ∼2.54%) and wax gourd (15.17% ∼17.79%). In the pumpkin, OGs were unevenly distributed across the 20 chromosomes, with the largest distribution in Chr1 and the least distribution in Chr19 (Figure 3B), with an approximately 1.59-fold difference. In the melon, OGs were unevenly distributed across the 12 chromosomes, with the largest distribution in Chr10 and the least distribution in Chr7 (Figure 3B), with an approximately 1.52-fold difference. Compared to pumpkin and melon, the spread of OGs on chromosomes was reasonably uniform in other Cucurbitaceae species. Besides, the distribution density of OGs was higher near the telomeres, and the distribution was relatively balanced on the chromosomes apart from the aggregation phenomenon in some chromosomal regions (Supplementary Figure 1).

### Orphan Genes Originating From Gene Duplication

**FIGURE 4:**
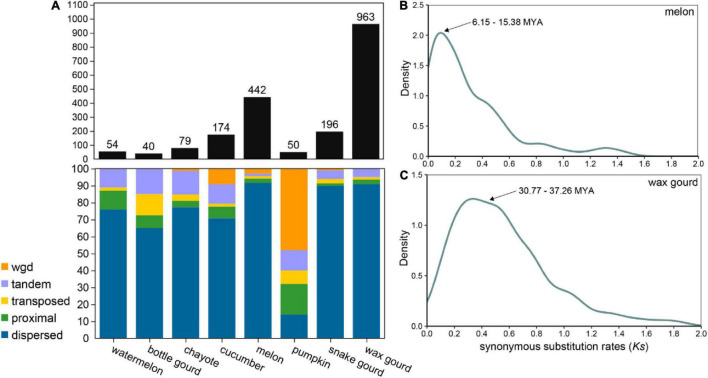
Orphan genes originating from gene duplication. **(A)** Statistics on the types of OGs originating from gene duplication in eight Cucurbitaceae species. **(B)** Density distribution of synonymous substitution rate (*Ks*) values between OGs and paralogous genes in melon. **(C)** Density distribution of *Ks* values between OGs and paralogous genes in wax gourd.

The evolutionary origin of OGs is a microevolutionary process in which the structure of a gene arises from the mutation of a germ cell gene, and the study of how genes arise and differentiate is essential to explain the generation and succession of novel phenotypes and eventually biodiversity (Tautz and Domazet-Lošo, 2011; Long et al., 2013). OGs are typically generated by multiple combinations of mechanisms of origin, and gene duplication is thought to be a major driving force of the production of OGs, for example, in *Arabidopsis thaliana*, rice, *Drosophila*, and primates (Guo et al., 2007; Toll-Riera et al., 2009; Donoghue et al., 2011). OGs originating from gene duplication were greatly varied among the eight Cucurbitaceae species, ranging from 50 (2%) in pumpkin 196 (37.05%) in snake gourd (Figure 4A and Supplementary Table 2). Most Cucurbitaceae species have a dispersed gene duplication type, except for pumpkin where the most dominant gene duplication type is whole genome duplication. We further estimated the duplication time of OGs in melon and wax gourd. We found that the duplication time of OGs was about 6.15∼15.38 MYA in melon, coinciding with the differentiation time of melon and cucumber (10 MYA), and maybe related to its correlated biologically relevant characters (Figure 4B; Garcia-Mas et al., 2012). In wax gourd, it was about 30.77∼37.26 MAY. This timing coincides with tribe Benincaseae, which was estimated to be distinct from the tribe *Momordiceae*, containing bitter gourd (36.1 MYA, Figure 4C; Xie et al., 2019). In addition, we also analyzed OGs originating from gene overlap, TE exaptation, and *de novo* models in Cucurbitaceae species (Supplementary Table 1). The results showed a large variation, and for the gene overlap originated OGs, ranging from 12 (1.91%) in chayote 103 (11.84%) in bottle gourd. For the TE exaptation originated OGs, ranging from 24 (1.04%) in pumpkin 129 (24.39%) in snake gourd. For the *de novo* originated OGs, ranging from 3 (0.57%) in snake gourd 106 (4.63%) in melon (Supplementary Table 2). Future studies will be needed to determine whether these OGs with different models of origin are functional and to reveal their relevance in the adaptive evolution and generation of new traits of agronomic importance of Cucurbitaceae species.

### Characterization of Expression Patterns of Orphan Genes

OGs are often functionally enigmatic due to the lack of homology and functional structural information. To reveal the potential biological functions of these OGs, we investigated the gene expression patterns based on transcriptome profiling data. In this study, we performed an analysis of the OGs expression based on previously published RNA-seq data in (1) five tissues in melon (root, leaf, male flower, female flower, and fruit); (2) six tissues in cucumber (root, stem, leaf, male flower, female flower, and ovary). Transcriptional data showed evidence of expression of 1,383 (60.47%) OGs and 24,358 (87.96%) NOGs in melon, and 937 (37.12%) OGs and 20,292 (96.82%) NOGs in cucumber. Further studies found, in melon and cucumber (Supplementary Table 4), there were 636 (27.81%) and 165 (17.61%) OGs, and 4,935 (17.82%) and 2,162 (10.65%) NOGs showed tissue-specific expression, respectively. It is evident that OGs were less abundantly expressed but had a higher tissue-specific expression, which is in agreement with the expression patterns of OGs observed in other species (Long et al., 2003; Wu et al., 2011) and these tissue-specific expressed genes may be associated with specific phenotypes or specific physiological processes. Interestingly, the largest proportion of these OGs was significantly more tissue-specific expressed in flower than in other tissues (melon: 69.97% and cucumber: 40.61%) (Supplementary Table 4), and more detectable expression was found in male flower (melon: 35.38% and cucumber: 21.82%) (Supplementary Table 4). Many studies have shown that OGs, or young genes as some researchers call them, are more inclined to be expressed in the male organs (McCarrey and Thomas, 1987; Yang et al., 2009; Cui et al., 2015; Ruiz-Orera et al., 2015).

### Functional Inference of Orphan Genes

**FIGURE 5:**
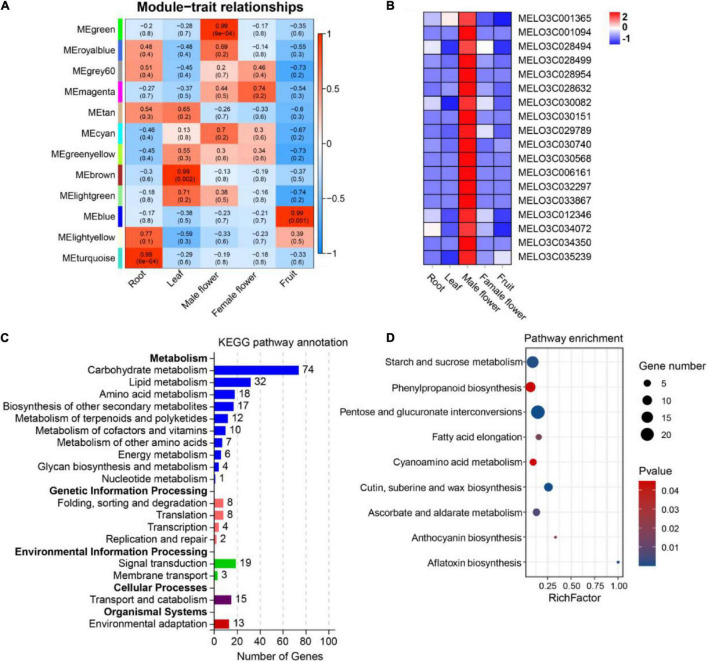
Expression patterns and functional prediction of OGs in different tissues of melon, includes root, leaf, male flower, female flower and fruit. **(A)** Gene significance map. **(B)** Heat map of OGs expression in different tissues inside the MEgreen module. **(C)** Functional annotation of KEGG for co-expressed genes of OGs. **(D)** KEGG enrichment analysis of co-expressed genes of OGs.

Using co-expressed genes to predict the function of OGs is an effective method (Li G. et al., 2019; Zhang et al., 2019; Ma et al., 2021). To investigate the underlying functions of OGs in melon, we used WGCNA analysis to identify 12 co-expressed gene modules (Figure 5A). After screening, 1017 genes were identified in MEgreen module (tissue-specific expression in male flower), including 18 OGs (Supplementary Table 5 and Figure 5B). Functional annotation showed that co-expressed genes of OGs are frequently involved in carbohydrate metabolism (Figure 5C). Carbohydrates play a key role in the development of male gametophytes, providing nutrition for normal growth and possibly acting as signaling molecules to influence development in the process (Clément and Audran, 1995). Many male sterile lines have been revealed to be involved in disorders of carbohydrate metabolism (Dorion et al., 1996; Zhang et al., 2010; Min et al., 2013). In addition, KEGG analysis showed the co-expressed genes of OGs are significantly enriched (*P*-value < 0.05) in pentose and glucuronate interconversions, cutin, suberine and wax biosynthesis, starch and sucrose metabolism, ascorbate and aldarate metabolism, phenylpropanoid biosynthesis, and anthocyanin biosynthesis (Figure 5D). Transcriptome analysis of the flower organ of male-sterile and fertile plants of *Allium sativum* revealed a certain number of differential genes related to anthocyanin biosynthesis (Shemesh-Mayer et al., 2015). The sucrose transporter gene *CsSUT1* of cucumber is expressed in the male flower, and down-regulation of *CsSUT1*-RNA interference (RNAi) expression can induce male sterility, a process that primarily affects starch and sucrose metabolism, and pentose and glucuronate interconversions (Sun et al., 2019). In addition, the anthocyanin biosynthesis, phenylpropanoid biosynthesis, and ascorbate and aldarate metabolism are engaged in the scavenging of reactive oxygen species in plants under adversity stress (Xu L. et al., 2017; Sharma et al., 2019). In the process of plant growth and development, due to nucleoplasmic genetic disharmony and various external adverse environments, excess reactive oxygen is produced, which leads to abnormal function of the cell membrane system and eventually causes male sterility (Fridovich, 1978; Li et al., 2004). The results of various studies shows that the potential functions of OGs specifically expressed in the male flower of melon are closely related to male sterility.

**FIGURE 6:**
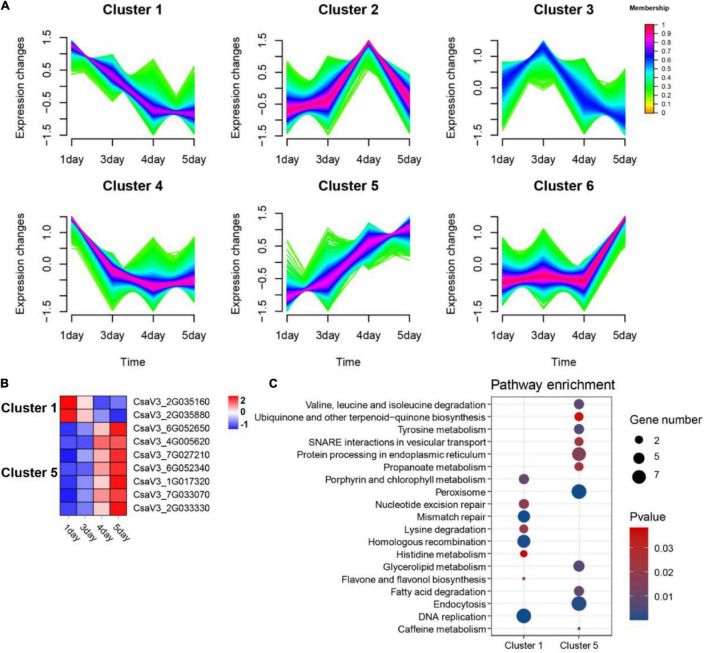
Functional prediction of OGs in the development of cucumber flowers. **(A)** Trends in the expression of differentially expressed genes at the stage of flower development. **(B)** Heat map of the expression of OGs under the trend of pattern Cluster 1 and Cluster 5. **(C)** KEGG enrichment analysis of co-expressed genes of OGs in Cluster 1 and Cluster 5.

To investigate the role of OGs on flower development, we analyzed transcriptome data from four different flowering stages of cucumber, including green bud (1 day), green-yellow bud (3 days), yellow bud (4 days), and flowering (5 days). Compared with the control group (green bud), a total of 10,248 significantly differentially expressed genes (DEGs) were identified, including 182 OGs. Fuzzy c-means clustering analysis of all DEGs (including OGs and NOGs) was further divided into six Clusters of gene co-expression patterns (Figure 6A). Genes with memberships > 0.6 in the Cluster were screened for subsequent functional enrichment analysis. There were a total of 385 DEGs in Cluster 1 (decreasing), including 2 OGs, and 381 DEGs in Cluster 5 (increasing), including 7 OGs (Figure 6B). KEGG enrichment results for co-expressed genes of OGs showed that DNA replication, Porphyrin and chlorophyll metabolism, and flavone and flavonol biosynthesis were significantly enriched in Cluster 1. Peroxisome, endocytosis, and tyrosine metabolism were significantly enriched in Cluster 3 (*P*-value < 0.05, Figure 6C). Peroxisomes are small cellular organelles that produce a variety of metabolites and are essential in modulating plant growth and development (Xu S. et al., 2017; Kao et al., 2018). *OsPEX5* encodes a peroxisome targeting sequence 1 (PTS1) receptor protein, whereas the mutants can cause abnormal rice spikelet morphology (You et al., 2019).

**FIGURE 7:**
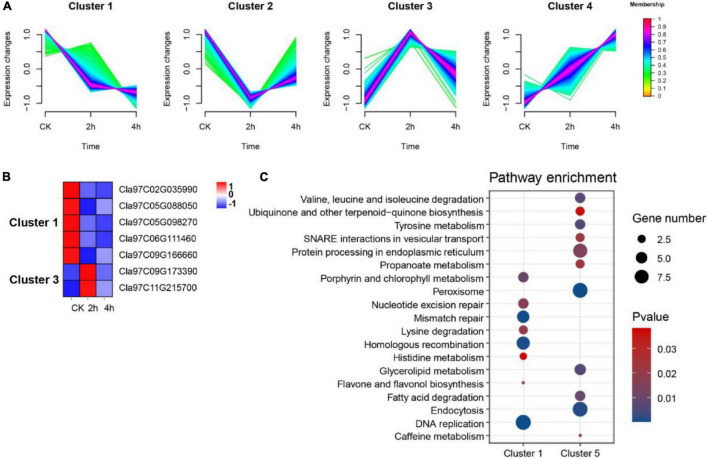
Functional prediction of OGs under osmotic stress. **(A)** Trends in the expression of differentially expressed genes at different time points under osmotic stress. **(B)** Heat map of the expression of OGs under the trend of pattern Cluster 1 and Cluster 3. **(C)** KEGG enrichment analysis of co-expressed genes of OGs in Cluster 1 and Cluster 3.

Evidence suggests that OGs are recruited into roles that regulate responses to changing environments (Gollery et al., 2006; Luhua et al., 2008). To probe the possible association between OGs and environmental adaptation in watermelon, we reanalyzed the expression of OGs in (1) suspension cells under osmotic stress (0, 2, and 4 h); (2) leaves under drought stress (4 days and 8 days); (3) roots and leaves under nitrogen (N) (LowN: 0.2 mM and HighN: 9 mM). In osmotic stress, compared with the control group (0 h), we identified 8 (up-regulated: 4, down-regulated: 4) and 3 down-regulated OGs in 2 h vs. 0h and 4 h vs. 0h were differentially expressed, respectively. A total of 9 OGs overlapped that were osmotic responsive (Supplementary Table 6). Fuzzy c-mean clustering analysis showed that all DEGs were classified into four Clusters (Figure 7A). The genes in the clusters with memberships > 0.6 were filtered out and subjected to KEGG enrichment analysis. There were a total of 1,049 DEGs in Cluster 1, including 5 OGs, and 423 DEGs in Cluster 3, including 2 OGs (Figure 7B). KEGG enrichment results for co-expressed genes of OGs showed that carbon fixation in photosynthetic organisms, Glycolysis/Gluconeogenesis, monoterpenoid biosynthesis, and zeatin biosynthesis pathway were significantly enriched in Cluster 1. Spliceosome, phosphatidylinositol signaling system, nitrogen metabolism, and plant hormone signal transduction were significantly enriched in Cluster 3 (*P*-value < 0.05, Figure 7C). In drought stress, a total of 4,090 DEGs were identified, of which 37 were OGs (Supplementary Table 6). A WGCNA for all DEGs associated with drought led to the further identification of two essential co-expression modules MEblack (drought treatment for 4 days) and MEturquoise (drought treatment for 8 days) (*P*-value < 0.05, Supplementary Figure 2), where 7 OGs were participating and enriched in GO terms, consisting ‘response to water’, ‘response to high light intensity’ and ‘response to hydrogen peroxide’ (*P*-value < 0.05, Supplementary Table 7). These correlations suggest a vehicle to infer the functions of OGs and indicate the incorporation of certain OGs into conserved stress networks as an underlying mechanism for watermelon to adapt to drought stress. In addition, we also identified three (Cla97C02G028900, Cla97C05G099800 and Cla97C09G167820) and two OGs (Cla97C06G126600 and Cla97C09G171550) that were differentially expressed in leaves and roots under nitrogen stress, respectively (Supplementary Table 6). Clearly, among the three abiotic stresses, OGs were more prominent under the drought condition.

The fruits of melons all undergo a process of small to large size, and wax gourd have larger fruits compared to other species in the Cucurbitaceae family. Xie et al. (2019) combined population genetics and linkage mapping to pinpoint 3,939 genes that may have been selected as part of domestication and improvement, with some possibly responsible for the large fruit size of wax gourd (Xie et al., 2019). Interestingly, by screening, we found 520 OGs (Supplementary Table 8), which represent about 13.2% (520/3939) of all genes domestication and improvement sweeps and about 11.44% (520/4546) of all OGs in the wax gourd genome.

In conclusion, we found that OGs played a crucial role in several aspects, such as male sterility, environmental adaptation and crop domestication. Our results provide the molecular basis and research direction for some important research mechanisms in Cucurbitaceae, but the specific functions of OGs need further experimental validation.

## Data Availability Statement

The original contributions presented in the study are included in the article/Supplementary Material, further inquiries can be directed to the corresponding author/s.

## Author Contributions

DM and FZ designed this experimental subject. DM were involved in the analysis of the entire study and drafted the manuscript. SL and ZZ performed the data collection. ZL, QD, KZ, KC, and FZ critically revised the manuscript. All authors read and approved the final manuscript.

## Conflict of Interest

The authors declare that the research was conducted in the absence of any commercial or financial relationships that could be construed as a potential conflict of interest.

## Publisher’s Note

All claims expressed in this article are solely those of the authors and do not necessarily represent those of their affiliated organizations, or those of the publisher, the editors and the reviewers. Any product that may be evaluated in this article, or claim that may be made by its manufacturer, is not guaranteed or endorsed by the publisher.

## Footnotes

1
http://cucurbitgenomics.org/

2
http://www.plantgdb.org/

3
https://phytozome-next.jgi.doe.gov/

4
https://www.uniprot.org/uniprot/

5
ftp://ftp.ncbi.nlm.nih.gov/

6
https://www.ncbi.nlm.nih.gov/

7
https://github.com/DongnaMa/python-for-bio

8
http://mg2c.iask.in/mg2cv2.0/

9
https://github.com/tanghaibao/bio-pipeline/tree/master/synonymous_calculation

10
https://doi.org/10.17632/wxdm2gvbn3.1

11
https://www.omicshare.com/

## Supplementary Material

Supplementary Figure 1 Chromosomal distribution of the identified OGs in eight Cucurbitaceae species. Black horizontal lines represent OGs. **(A)** watermelon, **(B)** bottle gourd, **(C)** chayote, **(D)** cucumber, **(E)** melon, **(F)** pumpkin, **(G)** snake gourd, **(H)** wax gourd.

Supplementary Figure 2 Gene significance map.

## References

[B1] Andrade M. A. O’Donoghue S. I. Rost B. (1998). Adaptation of protein surfaces to subcellular location. *J. Mol. Biol.* 276 517–525. 10.1006/jmbi.1997.1498 9512720

[B2] Arendsee Z. W. Li L. Wurtele E. S. (2014). Coming of age: orphan genes in plants. *Trends Plant Sci.* 19 698–708. 10.1016/j.tplants.2014.07.003 25151064

[B3] Campbell M. A. Zhu W. Jiang N. Lin H. Ouyang S. (2007). Identification and characterization of lineage-specific genes within the Poaceae. *Plant Physiol.* 145 1311–1322. 10.1104/pp.107.104513 17951464 PMC2151710

[B4] Chou K. C. Shen H. B. (2008). Cell-PLoc: a package of Web servers for predicting subcellular localization of proteins in various organisms. *Nat. Protoc.* 3 153–162. 10.1038/nprot.2007.494 18274516

[B5] Clément C. Audran J. C. (1995). Anther wall layers control pollen sugar nutrition in Lilium. *Protoplasma* 187 172–181. 10.1007/BF01280246

[B6] Cui X. Lv Y. Chen M. Nikoloski Z. Twell D. Zhang D. (2015). Young Genes out of the Male: an Insight from Evolutionary Age Analysis of the Pollen Transcriptome. *Mol. Plant* 8 935–945. 10.1016/j.molp.2014.12.008 25670339

[B7] Doerks T. Copley R. R. Schultz J. Ponting C. P. Bork P. (2002). Systematic identification of novel protein domain families associated with nuclear functions. *Genome Res.* 12 47–56. 10.1101/gr.203201 11779830 PMC155265

[B8] Domazet-Loso T. Tautz D. (2003). An evolutionary analysis of orphan genes in *Drosophila*. *Genome Res.* 13 2213–2219. 10.1101/gr.1311003 14525923 PMC403679

[B9] Donoghue M. T. Keshavaiah C. Swamidatta S. H. Spillane C. (2011). Evolutionary origins of Brassicaceae specific genes in *Arabidopsis thaliana*. *BMC Evol. Biol.* 11:47. 10.1186/1471-2148-11-47 21332978 PMC3049755

[B10] Dorion S. Lalonde S. Saini H. S. (1996). Induction of Male Sterility in Wheat by Meiotic-Stage Water Deficit Is Preceded by a Decline in Invertase Activity and Changes in Carbohydrate Metabolism in Anthers. *Plant Physiol.* 111 137–145. 10.1104/pp.111.1.137 12226280 PMC157820

[B11] Emanuelsson O. Brunak S. von Heijne G. Nielsen H. (2007). Locating proteins in the cell using TargetP, SignalP and related tools. *Nat. Protoc.* 2 953–971. 10.1038/nprot.2007.131 17446895

[B12] Fischer D. Eisenberg D. (1999). Finding families for genomic ORFans. *Bioinformatics* 15 759–762. 10.1093/bioinformatics/15.9.759 10498776

[B13] Fridovich I. (1978). The biology of oxygen radicals. *Science* 201 875–880. 10.1126/science.210504 210504

[B14] Garcia-Mas J. Benjak A. Sanseverino W. Bourgeois M. Mir G. González V. M. (2012). The genome of melon (*Cucumis melo* L.). *Proc. Natl. Acad. Sci. U. S. A.* 109 11872–11877. 10.1073/pnas.1205415109 22753475 PMC3406823

[B15] Gaut B. S. Morton B. R. McCaig B. C. Clegg M. T. (1996). Substitution rate comparisons between grasses and palms: synonymous rate differences at the nuclear gene Adh parallel rate differences at the plastid gene rbcL. *Proc. Natl. Acad. Sci. U. S. A.* 93 10274–10279. 10.1073/pnas.93.19.10274 8816790 PMC38374

[B16] Gollery M. Harper J. Cushman J. Mittler T. Girke T. Zhu J. K. (2006). What makes species unique? The contribution of proteins with obscure features. *Genome Biol.* 7:R57. 10.1186/gb-2006-7-7-r57 16859532 PMC1779552

[B17] Guo S. Zhao S. Sun H. Wang X. Wu S. Lin T. (2019). Resequencing of 414 cultivated and wild watermelon accessions identifies selection for fruit quality traits. *Nat. Genet.* 51 1616–1623. 10.1038/s41588-019-0518-4 31676863

[B18] Guo W. J. Li P. Ling J. Ye S. P. (2007). Significant comparative characteristics between orphan and nonorphan genes in the rice (*Oryza sativa* L.) genome. *Comp. Funct. Genomics* 2007:21676. 10.1155/2007/21676 18273382 PMC2216055

[B19] Guo Y. L. (2013). Gene family evolution in green plants with emphasis on the origination and evolution of *Arabidopsis thaliana* genes. *Plant J.* 73 941–951. 10.1111/tpj.12089 23216999

[B20] Hu Y. Ma D. Ning S. Ye Q. Zhao X. Ding Q. (2021). High-Quality Genome of the Medicinal Plant *Strobilanthes cusia* Provides Insights Into the Biosynthesis of Indole Alkaloids. *Front. Plant Sci.* 12:742420. 10.3389/fpls.2021.742420 34659312 PMC8515051

[B21] Hunsakunachai N. Nuengchamnong N. Jiratchariyakul W. Kummalue T. Khemawoot P. (2019). Pharmacokinetics of cucurbitacin B from *Trichosanthes cucumerina* L. in rats. *BMC Complement. Altern. Med.* 19:157. 10.1186/s12906-019-2568-7 31272429 PMC6609384

[B22] Kaessmann H. (2010). Origins, evolution, and phenotypic impact of new genes. *Genome Res.* 20 1313–1326. 10.1101/gr.101386.109 20651121 PMC2945180

[B23] Kao Y. T. Gonzalez K. L. Bartel B. (2018). Peroxisome Function, Biogenesis, and Dynamics in Plants. *Plant Physiol.* 176 162–177. 10.1104/pp.17.01050 29021223 PMC5761812

[B24] Khaldi N. Shields D. C. (2011). Shift in the isoelectric-point of milk proteins as a consequence of adaptive divergence between the milks of mammalian species. *Biol. Direct* 6:40. 10.1186/1745-6150-6-40 21801421 PMC3189186

[B25] Khalturin K. Anton-Erxleben F. Sassmann S. Wittlieb J. Hemmrich G. Bosch T. C. (2008). A novel gene family controls species-specific morphological traits in *Hydra*. *PLoS Biol.* 6:e278. 10.1371/journal.pbio.0060278 19018660 PMC2586386

[B26] Kiraga J. Mackiewicz P. Mackiewicz D. Kowalczuk M. Biecek P. Polak N. (2007). The relationships between the isoelectric point and: length of proteins, taxonomy and ecology of organisms. *BMC Genomics* 8:163. 10.1186/1471-2164-8-163 17565672 PMC1905920

[B27] Knowles D. G. McLysaght A. (2009). Recent *de novo* origin of human protein-coding genes. *Genome Res.* 19 1752–1759. 10.1101/gr.095026.109 19726446 PMC2765279

[B28] Li G. Wu X. Hu Y. Muñoz-Amatriaín M. Luo J. Zhou W. (2019). Orphan genes are involved in drought adaptations and ecoclimatic-oriented selections in domesticated cowpea. *J. Exp. Bot.* 70 3101–3110. 10.1093/jxb/erz145 30949664

[B29] Li L. Zheng W. Zhu Y. Ye H. Tang B. Arendsee Z. W. (2015). *QQS* orphan gene regulates carbon and nitrogen partitioning across species via NF-YC interactions. *Proc. Natl. Acad. Sci. U. S. A.* 112 14734–14739. 10.1073/pnas.1514670112 26554020 PMC4664325

[B30] Li Q. Li H. Huang W. Xu Y. Zhou Q. Wang S. (2019). A chromosome-scale genome assembly of cucumber (*Cucumis sativus* L.). *Gigascience* 8:giz072. 10.1093/gigascience/giz072 31216035 PMC6582320

[B31] Li S. Wan C. Kong J. Zhang Z. Li Y. Zhu Y. (2004). Programmed cell death during microgenesis in a Honglian CMS line of rice is correlated with oxidative stress in mitochondria. *Funct. Plant Biol.* 31 369–376. 10.1071/fp03224 32688907

[B32] Lin H. Moghe G. Ouyang S. Iezzoni A. Shiu S. H. Gu X. (2010). Comparative analyses reveal distinct sets of lineage-specific genes within *Arabidopsis thaliana*. *BMC Evol. Biol.* 10:41. 10.1186/1471-2148-10-41 20152032 PMC2829037

[B33] Lindskog C. Kuhlwilm M. Davierwala A. Fu N. Hegde G. Uhlén M. (2014). Analysis of Candidate Genes for Lineage-Specific Expression Changes in Humans and Primates. *J. Proteome Res.* 13 3596–3606. 10.1021/pr500045f 24911366

[B34] Long M. Betrán E. Thornton K. Wang W. (2003). The origin of new genes: glimpses from the young and old. *Nat. Rev. Genet.* 4 865–875. 10.1038/nrg1204 14634634

[B35] Long M. VanKuren N. W. Chen S. Vibranovski M. D. (2013). New gene evolution: little did we know. *Annu. Rev. Genet.* 47 307–333. 10.1146/annurev-genet-111212-133301 24050177 PMC4281893

[B36] Love M. I. Huber W. Anders S. (2014). Moderated estimation of fold change and dispersion for RNA-seq data with DESeq2. *Genome Biol.* 15:550. 10.1186/s13059-014-0550-8 25516281 PMC4302049

[B37] Luhua S. Ciftci-Yilmaz S. Harper J. Cushman J. Mittler R. (2008). Enhanced tolerance to oxidative stress in transgenic *Arabidopsis* plants expressing proteins of unknown function. *Plant Physiol.* 148 280–292. 10.1104/pp.108.124875 18614705 PMC2528079

[B38] Luhua S. Hegie A. Suzuki N. Shulaev E. Luo X. Cenariu D. (2013). Linking genes of unknown function with abiotic stress responses by high-throughput phenotype screening. *Physiol. Plant.* 148 322–333. 10.1111/ppl.12013 23517122

[B39] Ma D. Ding Q. Guo Z. Zhao Z. Wei L. Li Y. (2021). Identification, characterization and expression analysis of lineage-specific genes within mangrove species *Aegiceras corniculatum*. *Mol. Genet. Genomics* 296 1235–1247. 10.1007/s00438-021-01810-0 34363105

[B40] Ma L. Wang Q. Mu J. Fu A. Wen C. Zhao X. (2020). The genome and transcriptome analysis of snake gourd provide insights into its evolution and fruit development and ripening. *Hortic. Res.* 7:199. 10.1038/s41438-020-00423-9 33328440 PMC7704671

[B41] Ma S. Yuan Y. Tao Y. Jia H. Ma Z. (2020). Identification, characterization and expression analysis of lineage-specific genes within Triticeae. *Genomics* 112 1343–1350. 10.1016/j.ygeno.2019.08.003 31401233

[B42] McCarrey J. R. Thomas K. (1987). Human testis-specific *PGK* gene lacks introns and possesses characteristics of a processed gene. *Nature* 326 501–505. 10.1038/326501a0 3453121

[B43] Min L. Zhu L. Tu L. Deng F. Yuan D. Zhang X. (2013). Cotton GhCKI disrupts normal male reproduction by delaying tapetum programmed cell death via inactivating starch synthase. *Plant J.* 75 823–835. 10.1111/tpj.12245 23662698

[B44] Neme R. Tautz D. (2013). Phylogenetic patterns of emergence of new genes support a model of frequent *de novo* evolution. *BMC Genomics* 14:117. 10.1186/1471-2164-14-117 23433480 PMC3616865

[B45] Ni F. Qi J. Hao Q. Lyu B. Luo M. C. Wang Y. (2017). Wheat *Ms2* encodes for an orphan protein that confers male sterility in grass species. *Nat. Commun.* 8:15121. 10.1038/ncomms15121 28452349 PMC5414350

[B46] Pan J. B. Hu S. C. Wang H. Zou Q. Ji Z. L. (2012). PaGeFinder: quantitative identification of spatiotemporal pattern genes. *Bioinformatics* 28 1544–1545. 10.1093/bioinformatics/bts169 22492640 PMC3356841

[B47] Parker J. K. Hall L. O. (2014). Accelerating Fuzzy-C Means Using an Estimated Subsample Size. *IEEE Trans. Fuzzy Syst.* 22 1229–1244. 10.1109/tfuzz.2013.2286993 26617455 PMC4662382

[B48] Perochon A. Jianguang J. Kahla A. Arunachalam C. Scofield S. R. Bowden S. (2015). *TaFROG* Encodes a *Pooideae* Orphan Protein That Interacts with SnRK1 and Enhances Resistance to the Mycotoxigenic Fungus Fusarium graminearum. *Plant Physiol.* 169 2895–2906. 10.1104/pp.15.01056 26508775 PMC4677899

[B49] Qiao X. Li Q. Yin H. Qi K. Li L. Wang R. (2019). Gene duplication and evolution in recurring polyploidization–diploidization cycles in plants. *Genome Biol.* 20:38. 10.1186/s13059-019-1650-2 30791939 PMC6383267

[B50] Ruiz-Orera J. Hernandez-Rodriguez J. Chiva C. Sabidó E. Kondova I. Bontrop R. (2015). Origins of *De Novo* Genes in Human and Chimpanzee. *PLoS Genet.* 11:e1005721. 10.1371/journal.pgen.1005721 26720152 PMC4697840

[B51] Savojardo C. Martelli P. L. Fariselli P. Profiti G. Casadio R. (2018). BUSCA: an integrative web server to predict subcellular localization of proteins. *Nucleic Acids Res.* 46 W459–W466. 10.1093/nar/gky320 29718411 PMC6031068

[B52] Schaefer H. Renner S. S. (2011). Phylogenetic relationships in the order Cucurbitales and a new classification of the gourd family (*Cucurbitaceae*). *Taxon* 60 122–138. 10.1002/tax.601011

[B53] Sharma A. Shahzad B. Rehman A. Bhardwaj R. Landi M. Zheng B. (2019). Response of Phenylpropanoid Pathway and the Role of Polyphenols in Plants under Abiotic Stress. *Molecules* 24:2452. 10.3390/molecules24132452 31277395 PMC6651195

[B54] Shemesh-Mayer E. Ben-Michael T. Rotem N. Rabinowitch H. D. Doron-Faigenboim A. Kosmala A. (2015). Garlic (*Allium sativum* L.) fertility: transcriptome and proteome analyses provide insight into flower and pollen development. *Front. Plant Sci.* 6:271. 10.3389/fpls.2015.00271 25972879 PMC4411974

[B55] Sun H. Wu S. Zhang G. Jiao C. Guo S. Ren Y. (2017). Karyotype Stability and Unbiased Fractionation in the Paleo-Allotetraploid Cucurbita Genomes. *Mol. Plant* 10 1293–1306. 10.1016/j.molp.2017.09.003 28917590

[B56] Sun L. Sui X. Lucas W. J. Li Y. Feng S. Ma S. (2019). Down-regulation of the Sucrose Transporter CsSUT1 Causes Male Sterility by Altering Carbohydrate Supply. *Plant Physiol.* 180 986–997. 10.1104/pp.19.00317 30967482 PMC6548282

[B57] Sun W. Zhao X. W. Zhang Z. (2015). Identification and evolution of the orphan genes in the domestic silkworm, *Bombyx mori*. *FEBS Lett.* 589 2731–2738. 10.1016/j.febslet.2015.08.008 26296317

[B58] Tautz D. Domazet-Lošo T. (2011). The evolutionary origin of orphan genes. *Nat. Rev. Genet.* 12 692–702. 10.1038/nrg3053 21878963

[B59] Tay S. K. Blythe J. Lipovich L. (2009). Global discovery of primate-specific genes in the human genome. *Proc. Natl. Acad. Sci. U. S. A.* 106 12019–12024. 10.1073/pnas.0904569106 19581580 PMC2715485

[B60] Thümecke S. Beermann A. Klingler M. Schröder R. (2017). The flipflop orphan genes are required for limb bud eversion in the Tribolium embryo. *Front. Zool.* 14:48. 10.1186/s12983-017-0234-9 29075305 PMC5649079

[B61] Toll-Riera M. Bosch N. Bellora N. Castelo R. Armengol L. Estivill X. (2008). Origin of Primate Orphan Genes: a Comparative Genomics Approach. *Mol. Biol. Evol.* 26 603–612. 10.1093/molbev/msn281 19064677

[B62] Toll-Riera M. Bosch N. Bellora N. Castelo R. Armengol L. Estivill X. (2009). Origin of primate orphan genes: a comparative genomics approach. *Mol. Biol. Evol.* 26 603–612. 19064677 10.1093/molbev/msn281

[B63] Wissler L. Gadau J. Simola D. F. Helmkampf M. Bornberg-Bauer E. (2013). Mechanisms and Dynamics of Orphan Gene Emergence in Insect Genomes. *Genome Biol. Evol.* 5 439–455. 10.1093/gbe/evt009 23348040 PMC3590893

[B64] Wu D. D. Irwin D. M. Zhang Y. P. (2011). *De novo* origin of human protein-coding genes. *PLoS Genet.* 7:e1002379. 10.1371/journal.pgen.1002379 22102831 PMC3213175

[B65] Xia X. (2018). DAMBE7: new and Improved Tools for Data Analysis in Molecular Biology and Evolution. *Mol. Biol. Evol.* 35 1550–1552. 10.1093/molbev/msy073 29669107 PMC5967572

[B66] Xie D. Xu Y. Wang J. Liu W. Zhou Q. Luo S. (2019). The wax gourd genomes offer insights into the genetic diversity and ancestral cucurbit karyotype. *Nat. Commun.* 10:5158. 10.1038/s41467-019-13185-3 31727887 PMC6856369

[B67] Xu L. Zhao H. Ruan W. Deng M. Wang F. Peng J. (2017). ABNORMAL INFLORESCENCE MERISTEM1 Functions in Salicylic Acid Biosynthesis to Maintain Proper Reactive Oxygen Species Levels for Root Meristem Activity in Rice. *Plant Cell* 29 560–574. 10.1105/tpc.16.00665 28298519 PMC5385951

[B68] Xu S. He Z. Zhang Z. Guo Z. Guo W. Lyu H. (2017). The origin, diversification and adaptation of a major mangrove clade (*Rhizophoreae*) revealed by whole-genome sequencing. *Natl. Sci. Rev.* 4 721–734. 10.1093/nsr/nwx065 31258950 PMC6599620

[B69] Xu Y. Wu G. Hao B. Chen L. Deng X. Xu Q. (2015). Identification, characterization and expression analysis of lineage-specific genes within sweet orange (*Citrus sinensis*). *BMC Genomics* 16:995. 10.1186/s12864-015-2211-z 26597278 PMC4657247

[B70] Yan H. Zhang W. Lin Y. Dong Q. Peng X. Jiang H. (2014). Different evolutionary patterns among intronless genes in maize genome. *Biochem. Biophys. Res. Commun.* 449 146–150. 10.1016/j.bbrc.2014.05.008 24820954

[B71] Yang L. Zou M. Fu B. He S. (2013). Genome-wide identification, characterization, and expression analysis of lineage-specific genes within zebrafish. *BMC Genomics* 14:65. 10.1186/1471-2164-14-65 23368736 PMC3599513

[B72] Yang X. Jawdy S. Tschaplinski T. J. Tuskan G. A. (2009). Genome-wide identification of lineage-specific genes in Arabidopsis, Oryza and Populus. *Genomics* 93 473–480. 10.1016/j.ygeno.2009.01.002 19442640

[B73] Yin Y. Fischer D. (2006). On the origin of microbial ORFans: quantifying the strength of the evidence for viral lateral transfer. *BMC Evol. Biol.* 6:63. 10.1186/1471-2148-6-63 16914045 PMC1559721

[B74] Yin Y. Fischer D. (2008). Identification and investigation of ORFans in the viral world. *BMC Genomics* 9:24. 10.1186/1471-2164-9-24 18205946 PMC2245933

[B75] You X. Zhu S. Zhang W. Zhang J. Wang C. Jing R. (2019). OsPEX5 regulates rice spikelet development through modulating jasmonic acid biosynthesis. *New Phytol.* 224 712–724. 10.1111/nph.16037 31264225

[B76] Zhang G. Wang H. Shi J. Wang X. Zheng H. Wong G. K.-S. (2007). Identification and characterization of insect-specific proteins by genome data analysis. *BMC Genomics* 8:93. 10.1186/1471-2164-8-93 17407609 PMC1852559

[B77] Zhang H. Liang W. Yang X. Luo X. Jiang N. Ma H. (2010). Carbon starved anther encodes a MYB domain protein that regulates sugar partitioning required for rice pollen development. *Plant Cell* 22 672–689. 10.1105/tpc.109.073668 20305120 PMC2861464

[B78] Zhang J. (2003). Evolution by gene duplication: an update. *Trends Ecol. Evol.* 18 292–298. 10.1016/s0169-5347(03)00033-8

[B79] Zhang W. Gao Y. Long M. Shen B. (2019). Origination and evolution of orphan genes and *de novo* genes in the genome of Caenorhabditis elegans. *Sci. China Life Sci.* 62 579–593. 10.1007/s11427-019-9482-0 30919281

