# Supplementary figures and images for: Identification, Characterization and Function of Orphan Genes Among the Current Cucurbitaceae Genomes
Source: Front Plant Sci. 2022 May 4;13:872137. doi: 10.3389/fpls.2022.872137 (PMC9114813; doi:10.3389/fpls.2022.872137)

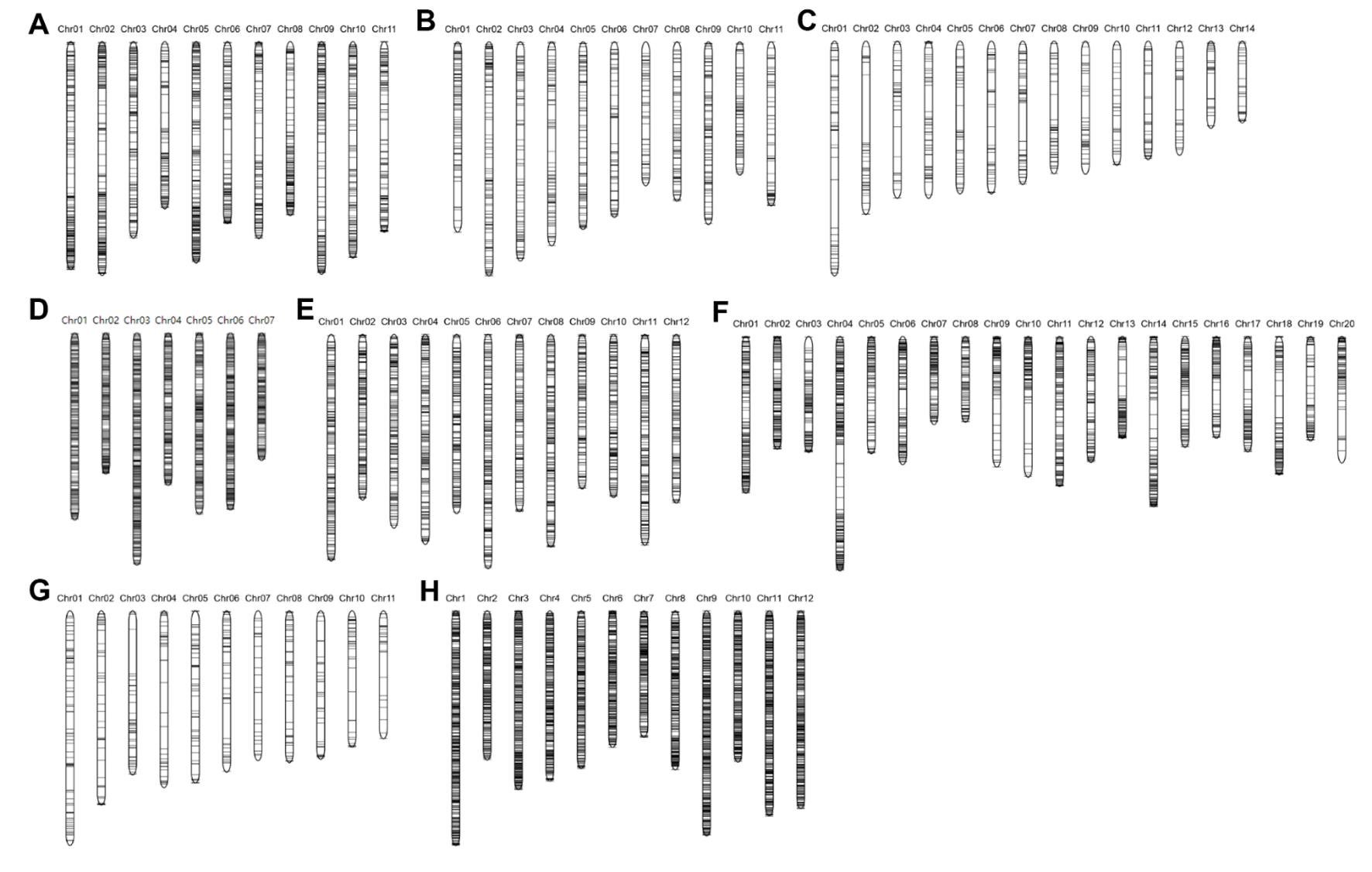

Supplement: Supplementary Figure 1 — Chromosomal distribution of the identified OGs in eight Cucurbitaceae species. Black horizontal lines represent OGs. (A) watermelon, (B) bottle gourd, (C) chayote, (D) cucumber, (E) melon, (F) pumpkin, (G) snake gourd, (H) wax gourd. [file Image_1.tif]

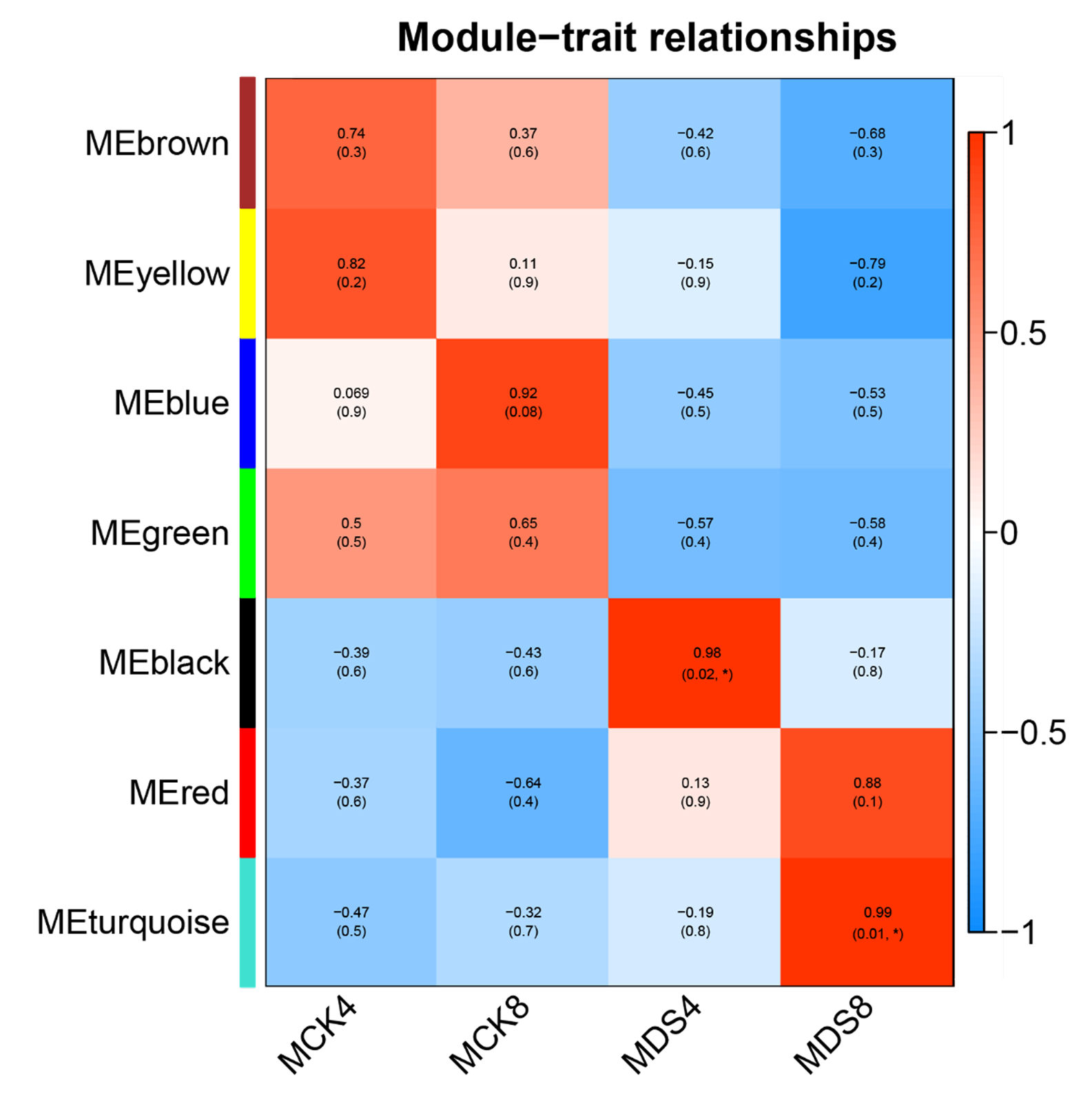

Supplement: Supplementary Figure 2 — Gene significance map. [file Image_2.tif]
