# Supplementary material for: Identification, Characterization and Function of Orphan Genes Among the Current Cucurbitaceae Genomes
Source: Front Plant Sci. 2022 May 4;13:872137. doi: 10.3389/fpls.2022.872137 (PMC9114813; doi:10.3389/fpls.2022.872137)
Supplement: Supplementary file 4 [file Table_2.DOCX]

| Species | watermelon | bottle gourd | chayote | cucumber | melon | pumpkin | snake gourd | wax gourd |
| --- | --- | --- | --- | --- | --- | --- | --- | --- |
| Number of genes | 22596 | 22472 | 38464 | 24317 | 29980 | 32205 | 32010 | 27467 |
| Number of OGs | 1652 | 870 | 627 | 2524 | 2287 | 2498 | 529 | 4547 |
| Percentage of OGs | 7.31% | 3.87% | 1.63% | 10.38% | 7.63% | 7.76% | 1.65% | 16.55% |
| Number of OGs originating from gene duplication | 54 | 40 | 79 | 174 | 442 | 50 | 196 | 963 |
| Percentage of OGs originating from gene duplication | 3.27% | 4.60% | 12.60% | 6.89% | 19.33% | 2.00% | 37.05% | 21.18% |
| Number of OGs originating from overlapping gene | 67 | 103 | 12 | 162 | 91 | 94 | 15 | 143 |
| Percentage of OGs originating from overlapping gene | 4.06% | 11.84% | 1.91% | 6.42% | 3.98% | 3.76% | 2.84% | 3.14% |
| Number of OGs originating from transposable element | 100 | 47 | 67 | 78 | 305 | 26 | 129 | 372 |
| Percentage of OGs originating from transposable element | 6.05% | 5.40% | 1.07% | 3.09% | 13.34% | 1.04% | 24.39% | 8.18% |
| Number of OGs originating from *de novo* | 48 | 11 | 4 | 105 | 106 | 19 | 3 | 61 |
| Percentage of OGs originating from *de novo* | 2.91% | 1.26% | 0.64% | 4.16% | 4.63% | 0.76% | 0.57% | 1.34% |

Table S2. Statistics of orphan genes (OGs) in eight Cucurbitaceae species
