# Supplementary material for: Identification, Characterization and Function of Orphan Genes Among the Current Cucurbitaceae Genomes
Source: Front Plant Sci. 2022 May 4;13:872137. doi: 10.3389/fpls.2022.872137 (PMC9114813; doi:10.3389/fpls.2022.872137)
Supplement: Supplementary file 5 [file Table_3.DOCX]

|  | Protein length (aa) | | Exons number | | Exon length | | Gene GC content (%) | | Isoelectric point | |
| --- | --- | --- | --- | --- | --- | --- | --- | --- | --- | --- |
|  | OGs | ECs | OGs | ECs | OGs | ECs | OGs | ECs | LSGs | ECs |
| watermelon | 95(84) | 411(342) | 2.64(2) | 5.45(4) | 108.78(90) | 226.88(129) | 37.53(36.93) | 38.86(37.68) | 8.94(9.52) | 7.62(7.44) |
| bottle gourd | 105(88) | 429(346) | 4(3) | 6.09(4) | 100.25(73) | 277.80(145) | 34.45(33.76) | 37.21(36.52) | 8.84(9.49) | 7.63(7.49) |
| chayote | 146(123) | 436 (365) | 2.77(2) | 8.09(5) | 289.38(141) | 295.8(153) | 38.47(37.18) | 38.25(37.45) | 8.97(9.93) | 7.6(7.41) |
| cucumber | 83(75) | 408(341) | 1.69(1) | 5.62(4) | 213.27(149) | 332.22(155) | 42.98(42.51) | 37.28(36.75) | 9.07 (9.84) | 7.62(7.46) |
| melon | 48(49) | 348(274) | 1.39(1) | 5.07(3) | 163.51(131) | 272.23(145) | 43.12(42.31) | 37.68(37.10) | 8.91(9.94) | 7.78(7.64) |
| pumpkin | 69(64) | 440(360) | 2.15(2) | 6.1(4) | 117.70(86) | 270.11(144) | 37.84(37.83) | 40.54(39.76) | 8.64(9.43) | 7.67(7.52) |
| snake gourd | 155(142) | 431(361) | 3.59(2) | 8.85(5) | 304.89(152) | 302.1(153) | 39.75(39.13) | 38.48(37.78) | 7.94(8.81) | 7.55(7.35) |
| wax gourd | 86(76) | 385(314) | 1.39(1) | 5.45(4) | 212.72(186) | 309.63(151) | 47.55(7.47) | 38.02(37.30) | 9.33(10) | 7.83(8) |

Table S3. Comparative structural statistics of orphan genes (OGs) and non-orphan genes (NOGs) in eight Cucurbitaceae species
