# Supplementary material for: Identification, Characterization and Function of Orphan Genes Among the Current Cucurbitaceae Genomes
Source: Front Plant Sci. 2022 May 4;13:872137. doi: 10.3389/fpls.2022.872137 (PMC9114813; doi:10.3389/fpls.2022.872137)
Supplement: Supplementary file 6 [file Table_4.DOCX]

| Items | Root | Stem | Leaf | Male flower | Female flower | Fruit | Ovary | Total |
| --- | --- | --- | --- | --- | --- | --- | --- | --- |
| Number of OGs (%) in melon | 88(13.84) |  | 41(6.45) | 225(35.38) | 220(34.59) | 62(9.75) |  | 636(100) |
| Number of NOGs (%) in melon | 1501(30.42) |  | 600(12.16) | 813(16.47) | 1386(28.09) | 635(12.87) |  | 4935(100) |
| Number of OGs (%) in cucumber | 29(17.58) | 14(8.48) | 26(15.76) | 36(21.82) | 31(18.79) |  | 29(17.58) | 165(100) |
| Number of NOGs (%) in cucumber | 1092(50.51) | 49(2.27) | 157(7.26) | 684(31.64) | 109(5.04) |  | 71(3.28) | 2162(100) |

Table S4. Tissue expression pattern of orphan genes (OGs) and non-orphan genes (NOGs) in melon and cucumber.
